# 1,8-Cineole Alleviates PA-Induced Lipid Accumulation, Oxidative Stress, and Inflammation via the TLR4/MyD88/NF-κB Signaling Pathway

**DOI:** 10.3390/molecules31111933

**Published:** 2026-06-03

**Authors:** Yanlong Li, Anning Zhan, Xiaobing Zhang, Yu Duan, Jiawen Tang, Hua Bai, Qi Wang

**Affiliations:** 1 School of Public Health, Kunming Medical University, Kunming 650500, China; 20230227@kmmu.edu.cn (Y.L.); 20220238@kmmu.edu.cn (A.Z.); 20240271@kmmu.edu.cn (X.Z.); 20230253@kmmu.edu.cn (Y.D.); 20240245@kmmu.edu.cn (J.T.); 2 Experiment Center for Medicial Science Research, Kunming Medical University, Kunming 650500, China

**Keywords:** 1,8-Cineole, lipid metabolism disorder, inflammation, oxidative stress, TLR4/MyD88/NF-κB signaling pathway

## Abstract

Objective: The present study investigates the effect of 1,8-cineole on improving lipid metabolism disorder by regulating oxidative stress and inflammation via the TLR4/MyD88/NF-κB pathway. 1,8-Cineole is a monoterpene compound widely found in the essential oils of many plants and has been reported to possess anti-inflammatory and antioxidant activities. However, its role in regulating lipid metabolism disorders remains unclear. This study aimed to investigate the effects of 1,8-cineole on lipid metabolism and explore the potential mechanisms related to oxidative stress and inflammation. Methods: Cell viability was assessed by MTT assay to determine the optimal PA concentration for inducing lipid accumulation and the non-cytotoxic range of 1,8-cineole in HepG2 and AML-12 cells. Lipid droplets were visualized by Oil Red O staining, while triglyceride (TG) and total cholesterol (TC) levels were quantified using enzymatic kits. Oxidative stress markers (ROS by DCFH-DA fluorescence; MDA by TBA method; CAT activity by ammonium molybdate method) and inflammatory cytokines (TNF-α, IL-6, IL-1β, IL-18 by ELISA) were measured. Western blotting analyzed key proteins in the TLR4/MyD88/NF-κB pathway (TLR4, MyD88, p-P65, p-IκBα). Pathway-specific inhibitors were employed for mechanistic validation. Results: 1,8-Cineole (up to 1000 μg/mL) showed no cytotoxicity. It significantly attenuated PA-induced lipid droplet accumulation, reduced TG and TC levels (*p* < 0.05), and ameliorated oxidative stress by decreasing ROS and MDA while enhancing CAT activity in AML-12 cells (*p* < 0.01). Furthermore, 1,8-cineole suppressed pro-inflammatory cytokine release (TNF-α, IL-6, and IL-1β; *p* < 0.01), whereas no significant effect was observed on IL-18 levels. Downregulated TLR4/MyD88/NF-κB pathway activation. Inhibition of TLR4 or NF-κB mirrored these protective effects. Conclusions: 1,8-Cineole alleviates PA-induced lipid metabolism disorders, oxidative stress, and inflammation in hepatocytes, likely through suppression of the TLR4/MyD88/NF-κB signaling pathway.

## 1. Introduction

Lipid metabolism disorder is a pathological condition characterised by the disruption of the dynamic balance in fat synthesis, transport, and catabolism, leading to abnormal blood lipid profiles and ectopic lipid deposition [1,2]. An imbalance of lipids and their metabolites during the stages of synthesis, storage, and catabolism has been demonstrated to alter the levels, structure, and function of plasma lipoproteins, thereby promoting the onset and progression of related diseases [3]. Lipid metabolism disorder is a common pathological basis for major chronic diseases such as obesity, metabolic dysfunction-associated steatotic liver disease (MASLD), and atherosclerosis. The fundamental factors that drive this condition include persistent inflammatory responses and oxidative stress-mediated imbalances in the synthesis, transport, and breakdown of lipids [4,5]. Current intervention strategies for lipid metabolism disorder-related diseases encompass lifestyle modifications, drug therapies such as orlistat, and surgical treatments. However, the effectiveness of lifestyle interventions is limited, and orlistat treatment can cause significant gastrointestinal adverse reactions with noticeable side effects [6]. In recent years, natural compounds have emerged as candidates for preventing lipid metabolism disorder-related diseases due to their diverse biological activities and relatively low side effects.

1,8-Cineole, a monoterpenoid compound, is considered to be one of the main components of plants and their essential oils. As a result of our research group’s investigations, the presence of this compound was previously identified in the essential oil of *Elsholtzia bodinieri* Vaniot. 1,8-Cineole is a versatile compound that finds application in a variety of fields, including the pharmaceutical and food industries. It is employed in products such as nasal sprays and disinfectants, as well as in the flavouring of food [7]. As demonstrated in [8], the addition of essential oils derived from plants, which contain 1,8-cineole, to foodstuffs and beverages can serve the purpose of preservatives. These effects are closely related to its potent antioxidant and anti-inflammatory properties. Research has demonstrated that 1,8-cineole is capable of manifesting antioxidant effects through the activation of nuclear factor erythroid 2-related factor 2 (Nrf2) and the upregulation of the expression of heme oxygenase-1 (HO-1), superoxide dismutase (SOD), and glutathione peroxidase (GPx) [9,10]. In a model of lead-induced liver injury, 1,8-cineole has been observed to increase hepatic SOD and GPx activities and significantly reduce malondialdehyde (MDA) levels [11]. In relation to its anti-inflammatory properties, 1,8-cineole has been observed to reduce the expression of inflammatory factors, including IL-1β and TNF-α, in lymphocytes [12]. Furthermore, the inhibition of NF-κB upstream signalling pathways, including extracellular signal-regulated kinase (ERK) and glycogen synthase kinase-3 (GSK-3), was observed [13]. In addition, the activity of mitogen-activated protein kinase (MAPK), protein kinase B (PKB), and triggering receptor expressed on myeloid cells-1 (TREM-1) was modulated, effectively suppressing the secretion of inflammatory cytokines [14,15]. Nevertheless, the direct effects of 1,8-cineole in models of lipid metabolism disorder remain to be elucidated. The present study systematically evaluated the effects of 1,8-cineole on lipid accumulation, inflammatory status, oxidative damage, and key protein expression in models of high-fat induced by palmitic acid, utilising HepG2 and AML-12 cells. In order to clarify its targets and signalling pathways, pathway inhibitors were employed for reverse validation. The objective of this research is to elucidate the mechanism.

However, the direct effects and underlying mechanisms of 1,8-cineole on lipid metabolism disorders remain unclear. Therefore, this study aimed to investigate the therapeutic potential of 1,8-cineole against PA-induced lipid dysregulation in hepatocytes and to elucidate whether its protective effects are mediated through the inhibition of the TLR4/MyD88/NF-κB pathway.

## 2. Results

### 2.1. Effects of 1,8-Cineole and PA on the Viability of HepG2 and AML12 Cells

**Figure 1:**
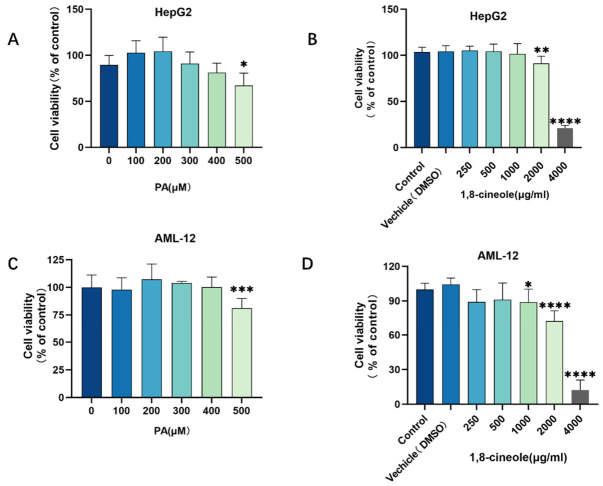
Effects of 1,8-Cineole and PA on the viability of HepG2 and AML12 cells: (**A**) Effect of PA on HepG2 cell viability (48 h). (**B**) Effect of 1,8-Cineole on HepG2 cell viability (48 h). (**C**) Effect of PA on AML12 cell viability (48 h). (**D**) Effect of 1,8-Cineole on AML12 cell viability (48 h). * indicates *p* < 0.05 compared to the control group; ** indicates *p* < 0.01 compared to the control group; *** indicates *p* < 0.001 compared to the control group, **** indicates *p* < 0.0001 compared to the control group.

The MTT assay results demonstrated that PA concentrations ≤ 400 µM did not exhibit significant effects on the viability of either cell type (*p* > 0.05). Conversely, a concentration of 500 μM significantly inhibited cell proliferation (*p* < 0.05) (Figure 1A,C). Consequently, a concentration of 400 μM PA with a 48-h treatment period was selected to establish the steatosis model.

With regard to 1,8-cineole, concentrations exceeding 1000 μg/mL significantly reduced cell viability in both HepG2 and AML12 cells (*p* < 0.05) (Figure 1B,D). The IC50 values of 1,8-cineole were estimated to be 3119 μg/mL (20,220 μM) for HepG2 cells and 2500 μg/mL (16,208 μM) for AML12 cells, indicating relatively low cytotoxicity within the tested concentration range. These IC50 values are comparable to those reported in other cell lines, where 1,8-cineole generally exhibits low cytotoxic activity at moderate concentrations. Consequently, three non-toxic concentrations of 1,8-cineole (250, 500, and 1000 μg/mL; corresponding to 1621, 3243, and 6486 μM, respectively) were selected for subsequent experiments. The corresponding final DMSO concentrations in the culture medium were approximately 0.06%, 0.12%, and 0.23% (*v*/*v*), respectively.

### 2.2. Effects of 1,8-Cineole on PA-Induced Lipid Metabolism Dysregulation in HepG2 and AML12 Cells

**Figure 2:**
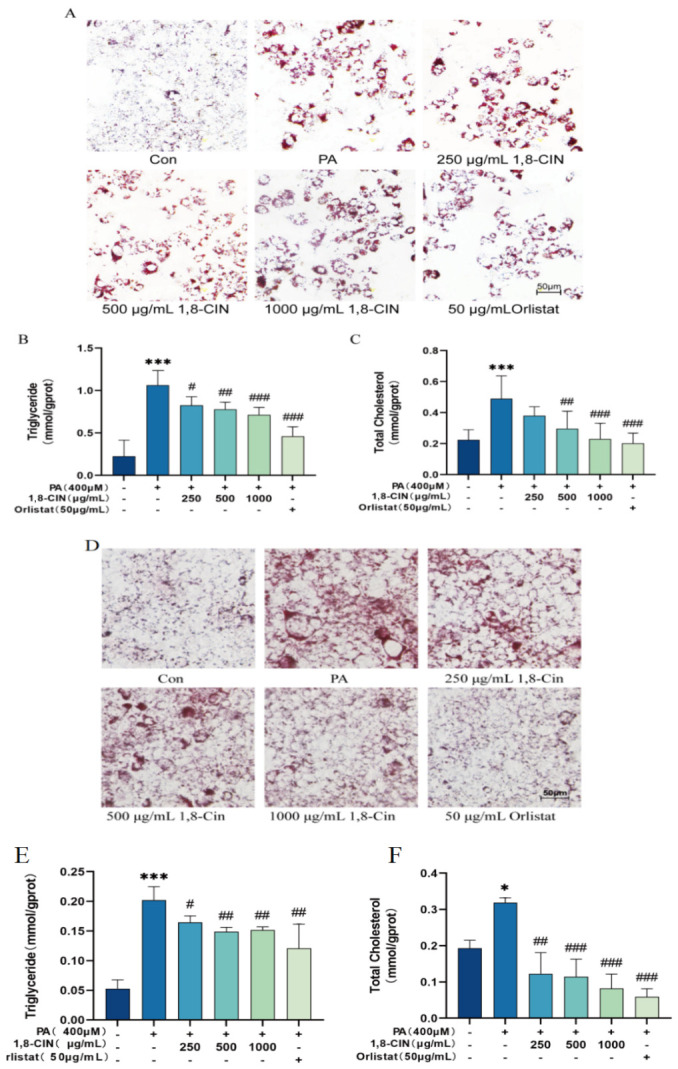
Effects of 1,8-Cineole on Oil Red O Staining, TG, and TC Levels in PA-Induced HepG2 and AML12 Cells: (**A**) Representative images (20× magnification) of Oil Red O staining in HepG2 cells; (**B**) Effect of 1,8-cineole on intracellular TG content in HepG2 cells; (**C**) Effect of 1,8-cineole on intracellular TC content in HepG2 cells; (**D**) Representative images (20× magnification) of Oil Red O staining in AML-12 cells; (**E**) Effect of 1,8-cineole on intracellular TG content in AML-12 cells; (**F**) Effect of 1,8-cineole on intracellular TC content in AML-12 cells. * *p* < 0.05 vs. control group; *** *p* < 0.001 vs. control group; ^#^ *p* < 0.05 vs. high-fat model group; ^##^ *p* < 0.01 vs. high-fat model group; ^###^ *p* < 0.001 vs. high-fat model group.

The results of Oil Red O staining and lipid quantification revealed that only a small number of lipid droplets were observed in the control group, while the PA-treated groups exhibited significant intracellular lipid droplet accumulation in both cell lines. Following intervention with 1,8-cineole, a decrease in lipid droplet deposition was observed in both cell lines (Figure 2A,D). In comparison with the control group, the levels of triglyceride (TG) and total cholesterol (TC) were significantly elevated in the model groups. However, following treatment with 1,8-cineole, a significant decrease in the levels of TC and TG was observed, with the magnitude of this decrease increasing with increasing concentrations of the intervention. It is noteworthy that at concentrations exceeding 500 μg/mL, the TC content exhibited a concentration-dependent decline (Figure 2B,C,E,F).

### 2.3. Effects of 1,8-Cineole on PA-Induced Oxidative Stress in HepG2 and AML12 Cells

**Figure 3:**
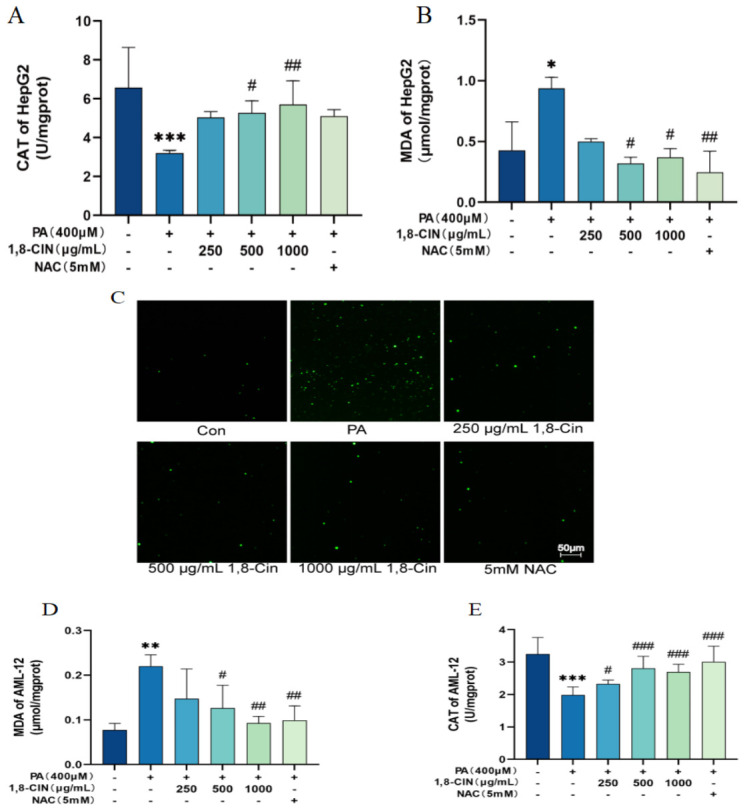
Effects of 1,8-D-Eucalyptol on PA-Induced Oxidative Stress in HepG2 and AML12 Cells. (**A**) Effect of 1,8-cineole on catalase (CAT) activity in HepG2 cells; (**B**) Effect of 1,8-cineole on malondialdehyde (MDA) content in HepG2 cells; (**C**) Effect of 1,8-cineole on reactive oxygen species (ROS) levels in AML-12 cells; (**D**) Effect of 1,8-cineole on MDA content in AML-12 cells; (**E**) Effect of 1,8-cineole on CAT activity in AML-12 cells. * *p* < 0.05 vs. control group; ** *p* < 0.01 vs. control group; *** *p* < 0.001 vs. control group. ^#^ *p* < 0.05 vs. high-fat model group; ^##^ *p* < 0.01 vs. high-fat model group; ^###^ *p* < 0.001 vs. high-fat model group.

In HepG2 cells (Figure 3A,B), there was a significant disruption to the redox balance following PA exposure, as evidenced by a decrease in CAT activity (*p* < 0.001) and an increase in MDA content (*p* < 0.05). The administration of 1,8-cineole resulted in a dose-dependent alleviation of this damage, as evidenced by a significant elevation in CAT levels (*p* < 0.05) and a reduction in MDA content (*p* < 0.05). In a similar manner, in AML-12 cells (Figure 3D,E), PA exposure resulted in a disturbance of the redox homeostasis, which led to a reduction in CAT levels (*p* < 0.001) and an increase in MDA content (*p* < 0.01). The use of a 2′,7′-dichlorodihydrofluorescein diacetate (DCFH-DA) fluorescent probe revealed that PA stimulation resulted in a significant increase in the intracellular ROS fluorescence intensity of AML-12 cells (*p* < 0.01). This increase was effectively scavenged by 1,8-cineole in a dose-dependent manner, as illustrated in Figure 3C.

### 2.4. Effects of 1,8-Cineole on PA-Induced Inflammatory Cytokine Secretion in AML-12 Cells

**Figure 4:**
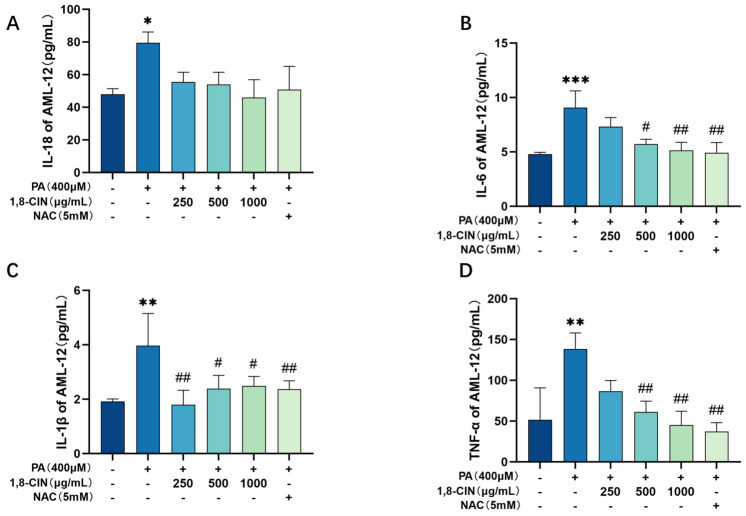
Effects of 1,8-Cineole on PA-Induced Inflammatory Cytokines in AML-12 Cells. (**A**) Effect of 1,8-cineole on IL-18 content in AML-12 cells; (**B**) Effect of 1,8-cineole on IL-6 content in AML-12 cells; (**C**) Effect of 1,8-cineole on IL-1β content in AML-12 cells; (**D**) Effect of 1,8-cineole on TNF-α content in AML-12 cells. * *p* < 0.05 vs. control group; ** *p* < 0.01 vs. control group; *** *p* < 0.001 vs. control group. ^#^ *p* < 0.05 vs. high-fat model group; ^##^ *p* < 0.01 vs. high-fat model group.

PA-induced AML-12 cells exhibited inflammatory responses, with a consistent upward trend in IL-1β expression (*p* < 0.01). Different concentrations of 1,8-cineole reduced IL-1β expression (*p* < 0.01), and this reduction was comparable to the effect of NAC (Figure 4C). Furthermore, varying concentrations of 1,8-menthol reversed PA-induced increases in IL-6 and TNF-α inflammatory factor levels (*p* < 0.01), exhibiting effects comparable to the antioxidant NAC. This anti-inflammatory effect demonstrated a dose-dependent response (Figure 4B,D). In contrast, PA had no significant effect on IL-18, and 1,8-cineole did not influence IL-18 expression (*p* > 0.05) (Figure 4A).

### 2.5. Effects of 1,8-Cineole on the Protein Expression Levels of the TLR4/MyD88/NF-κB Signaling Pathway in PA-Induced AML-12 Cells

**Figure 5:**
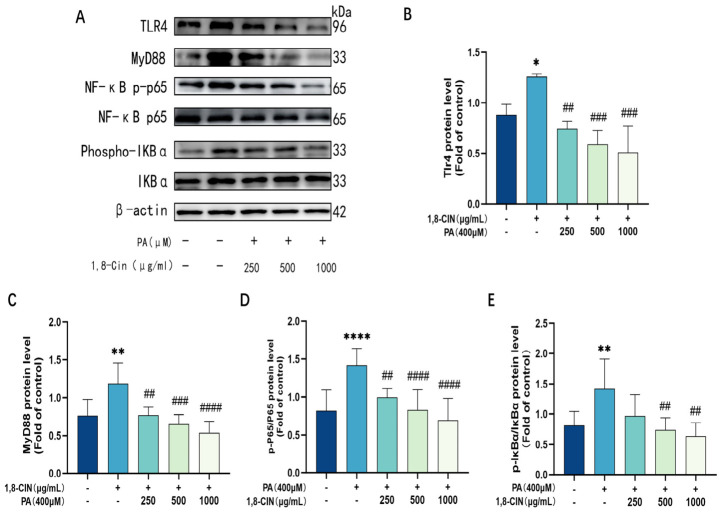
Effects of 1,8-Cineole on the Protein Expression of TLR4, MyD88, and NF-κB Signaling Pathway-Related Proteins in PA-Induced AML-12 Cells. (**A**) Representative immunoblots showing TLR4, MyD88, p-P65, P65, p-IκBα, and IκBα protein levels in AML-12 cells; (**B**) Relative expression level of TLR4 protein; (**C**) Relative expression level of MyD88 protein; (**D**) Relative expression ratio of p-P65/P65 protein; (**E**) Relative expression ratio of p-IκBα/IκBα protein. * *p* < 0.05 vs. control group; ** *p* < 0.01 vs. control group; **** *p* < 0.0001 vs. control group. ^##^ *p* < 0.01 vs. high-fat model group; ^###^ *p* < 0.001 vs. high-fat model group; ^####^ *p* < 0.0001 vs. high-fat model group.

Results showed that 1,8-cineole at 250 μg/mL inhibited PA-induced upregulation of TLR4 expression (*p* < 0.05) with a dose-dependent relationship (Figure 5A,B). At 500 μg/mL, 1,8-cineole inhibited expression of downstream MyD88, phosphorylated p65, and IKBα (*p* < 0.01) (Figure 5A,C,D,E).

### 2.6. Effects of TLR4 and NF-κB Inhibitors on Lipid Accumulation in the High-Fat Model of AML-12 Cells

**Figure 6:**
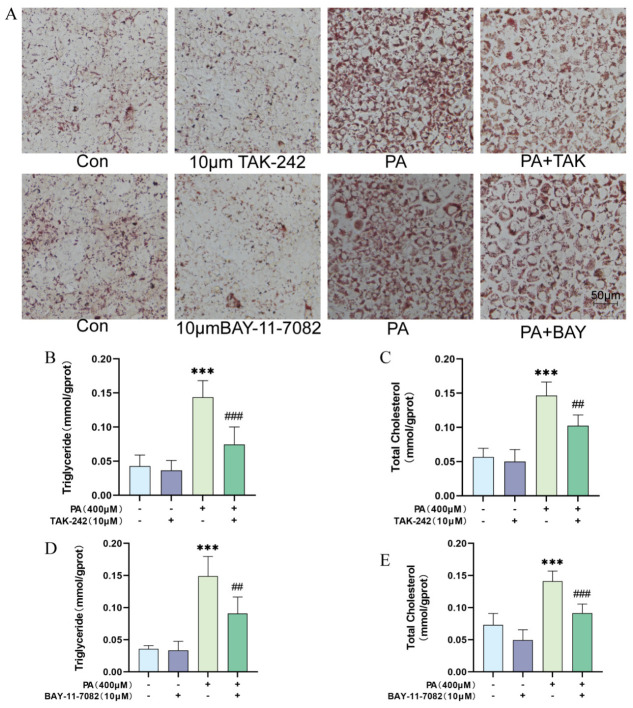
Effects of TAK-242 and BAY 11-7082 on Oil Red O Staining, TG, and TC in AML-12 Cells. (**A**) Representative images (20× magnification) of Oil Red O staining in AML-12 cells treated with TAK-242 or BAY 11-7082; (**B**) Effect of TAK-242 on TG levels in AML-12 cells; (**C**) Effect of TAK-242 on TC levels in AML-12 cells; (**D**) Effect of BAY 11-7082 on TG levels in AML-12 cells; (**E**) Effect of BAY 11-7082 on TC levels in AML-12 cells. *** *p* < 0.001 vs. control group; ^##^ *p* < 0.01 vs. high-fat model group; ^###^ *p* < 0.001 vs. high-fat model group.

As shown in Figure 6, compared with the control group, PA increased the number of intracellular lipid droplets as well as TG and TC content (*p* < 0.001). Inhibition with 10 μmol/L BAY 11-7082 or TAK-242 suppressed PA-induced increases in lipid droplet number and inhibited TG and TC expression (*p* < 0.01). However, no statistically significant differences in lipid droplet number or TG/TC content were observed between the Control group and groups treated with BAY 11-7082 or TAK-242 alone.

### 2.7. Effects of TLR4 and NF-κB Inhibitors on PA-Induced Oxidative Stress in AML-12 Cells

**Figure 7:**
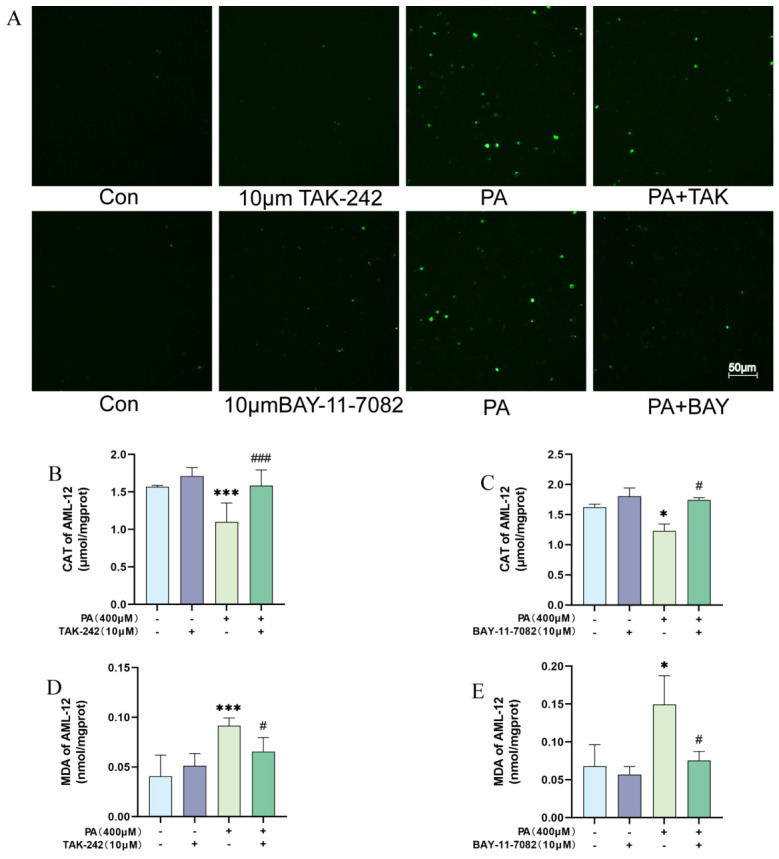
Effects of TAK-242 and BAY 11-7082 on Oxidative Stress in AML-12 Cells. (**A**) Effect of inhibitor treatment on ROS content in AML-12 cells; (**B**) Effect of TAK-242 on CAT activity in AML-12 cells; (**C**) Effect of BAY 11-7082 on CAT activity in AML-12 cells; (**D**) Effect of TAK-242 on MDA content in AML-12 cells; (**E**) Effect of BAY 11-7082 on MDA content in AML-12 cells. * *p* < 0.05 vs. control group; *** *p* < 0.001 vs. control group. ^#^ *p* < 0.05 vs. high-fat model group; ^###^ *p* < 0.001 vs. high-fat model group.

Compared with the Control group, PA significantly activated intracellular ROS expression (*p* < 0.01). The BAY 11-7082 inhibitor (10 μmol/L) or TAK-242 inhibitor effectively suppressed the PA-induced increase in ROS levels (*p* < 0.05) (Figure 7A). Furthermore, the reduction in CAT levels induced by PA treatment (*p* < 0.01) was reversed by intervention with 10 μmol/L BAY 11-7082 or TAK-242 (*p* < 0.05) (Figure 7B,C). Moreover, 10 μmol/L BAY 11-7082 or TAK-242 inhibition suppressed PA-induced MDA accumulation (*p* < 0.05) (Figure 7D,E).

### 2.8. Effects of TLR4 and NF-κB Inhibitors on PA-Induced Inflammatory Response in AML-12 Cells

**Figure 8:**
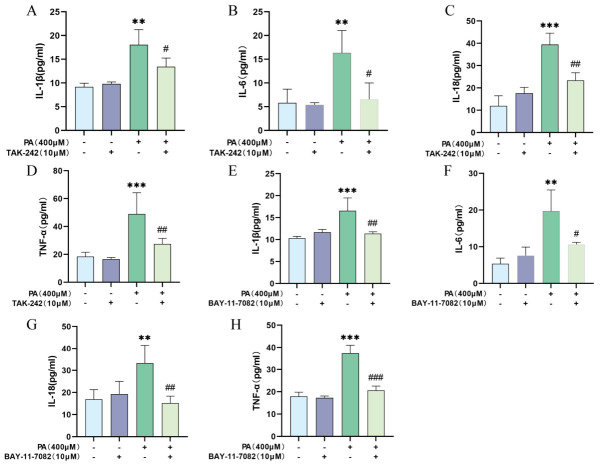
Effects of TAK-242 and BAY 11-7082 on Inflammatory Cytokines in AML-12 Cells. (**A**) Effect of TAK-242 on IL-1β content in AML-12 cells; (**B**) Effect of TAK-242 on IL-6 content in AML-12 cells; (**C**) Effect of TAK-242 on IL-18 content in AML-12 cells; (**D**) Effect of TAK-242 on TNF-α content in AML-12 cells; (**E**) Effect of BAY 11-7082 on IL-1β content in AML-12 cells; (**F**) Effect of BAY 11-7082 on IL-6 content in AML-12 cells; (**G**) Effect of BAY 11-7082 on IL-18 content in AML-12 cells; (**H**) Effect of BAY 11-7082 on TNF-α content in AML-12 cells. ** *p* < 0.01 vs. control group; *** *p* < 0.001 vs. control group. ^#^ *p* < 0.05 vs. high-fat model group; ^##^ *p* < 0.01 vs. high-fat model group; ^###^ *p* < 0.001 vs. high-fat model group.

Results showed that following PA treatment, AML-12 cells exhibited significantly elevated levels of TNF-α, IL-1β, IL-6, and IL-18 in cell culture supernatants compared to the control group (*p* < 0.01 or *p* < 0.001). Intervention with TLR4 inhibitors and NF-κB inhibitors reduced PA-induced secretion of inflammatory cytokines in AML-12 cells (*p* < 0.05 or *p* < 0.01). However, inflammatory levels in cells treated with TAK-242 inhibitor or BAY 11-7082 inhibitor alone showed no difference compared to the blank control group (Figure 8).

### 2.9. Effects of TLR4 and NF-κB Inhibitors on the Protein Expression of the TLR4/NF-κB Signaling Pathway in PA-Induced AML-12 Cells

**Figure 9:**
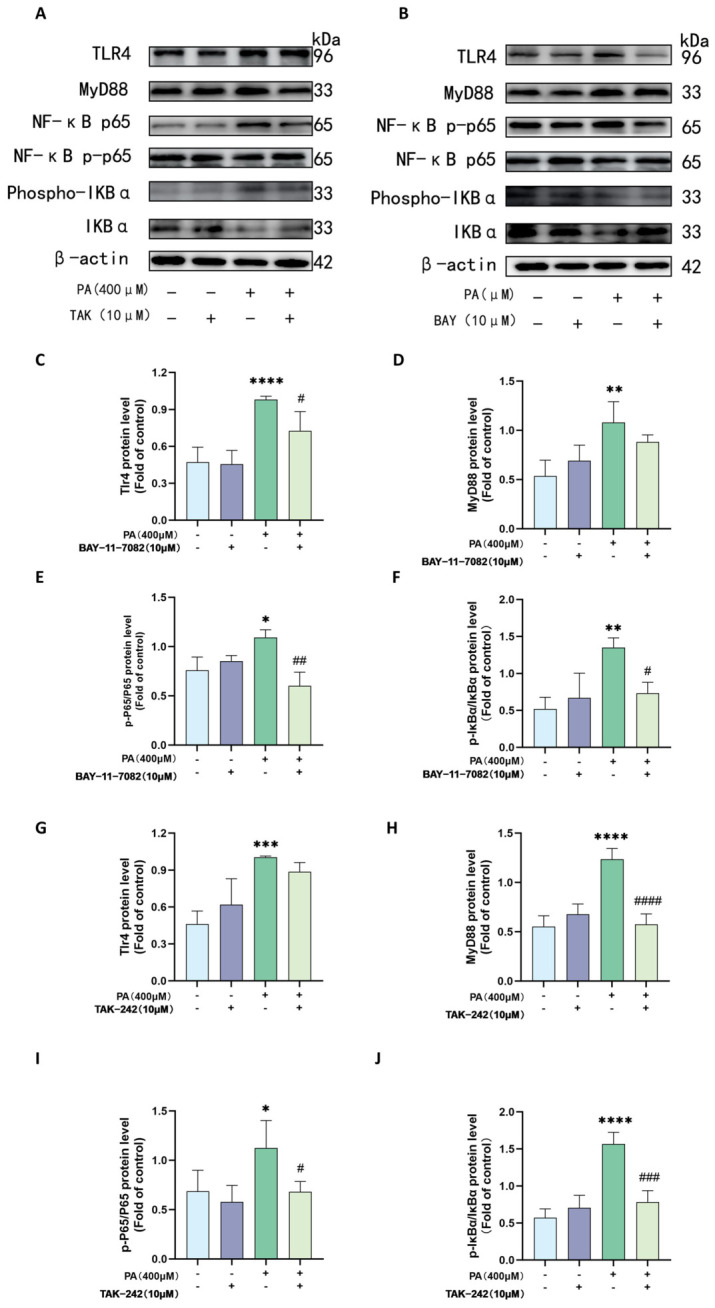
Effects of TAK-242 and BAY 11-7082 on the Protein Expression of TLR4, MyD88, and NF-κB Signaling Pathway-Related Proteins in AML-12 Cells. (**A**) Representative immunoblots showing TLR4, MyD88, p-P65, P65, p-IκBα, and IκBα protein levels in AML-12 cells treated with TAK-242; (**B**) Representative immunoblots showing TLR4, MyD88, p-P65, P65, p-IκBα, and IκBα protein levels in AML-12 cells treated with BAY 11-7082; (**C**) Effect of BAY 11-7082 on the relative expression level of TLR4 protein in AML-12 cells; (**D**) Effect of BAY 11-7082 on the relative expression level of MyD88 protein in AML-12 cells; (**E**) Effect of BAY 11-7082 on the relative expression ratio of p-P65/P65 protein in AML-12 cells; (**F**) Effect of BAY 11-7082 on the relative expression ratio of p-IκBα/IκBα protein in AML-12 cells; (**G**) Effect of TAK-242 on the relative expression level of TLR4 protein in AML-12 cells; (**H**) Effect of TAK-242 on the relative expression level of MyD88 protein in AML-12 cells; (**I**) Effect of TAK-242 on the relative expression ratio of p-P65/P65 protein in AML-12 cells; (**J**) Effect of TAK-242 on the relative expression ratio of p-IκBα/IκBα protein in AML-12 cells. * *p* < 0.05 vs. control group; ** *p* < 0.01 vs. control group; *** *p* < 0.001 vs. control group; **** *p* < 0.0001 vs. control group. ^#^ *p* < 0.05 vs. high-fat model group; ^##^ *p* < 0.01 vs. high-fat model group; ^###^ *p* < 0.001 vs. high-fat model group; ^####^ *p* < 0.0001 vs. high-fat model group.

As shown in Figure 9, when TAK-242/BAY 11-7082 inhibitors were administered alone in the blank group, there was no significant difference in the expression of signaling pathway proteins compared to the control group. However, as seen in (Figure 9C–J), after 48 h of intervention with 400 μM PA, the intracellular levels of TLR4, MyD88, p-P65/P65, and p-IKBα/IKBα showed an upward trend (*p* < 0.01). Furthermore, pretreatment with TLR4 antagonists not only suppressed PA-induced expression of TLR4 and its signaling molecule MyD88 in AML-12 cells (*p* < 0.01) (Figure 9C,D), but also suppressed the downstream p-P65/P65 and p-IKBα/IKBα protein levels (*p* < 0.01) (Figure 9E,F). In contrast, pretreatment with the BAY 11-7082 inhibitor had no significant effect on PA-induced increases in TLR4 and MyD88 protein levels (Figure 9G,H). However, it inhibited PA-induced increases in p-P65/P65 and p-IKBα/IKBα protein levels (*p* < 0.05 or *p* < 0.01).

## 3. Discussion

Lipid metabolism is a complex physiological process that plays a crucial role in regulating nutrient utilisation, hormone signalling, and body homeostasis [16]. Abnormalities in lipids and their metabolites during synthesis, storage, and catabolism frequently result in alterations to plasma lipoprotein levels, structure, and function, thereby contributing to the onset and progression of related diseases [17]. Abnormalities in the anabolism or catabolism of lipids within the body are termed lipid metabolism disorders, and their occurrence may be associated with individual lifestyle habits, poor dietary and exercise patterns, and metabolic abnormalities [18]. In recent years, there has been an observed increase in the incidence of lipid metabolism disorders and associated diseases [19]. It is imperative to recognise the significance of lipid homeostasis in ensuring the optimal functioning of cells and the body as a whole. This fundamental aspect serves as the cornerstone for sustaining normal physiological processes in living organisms. Disorders arising from an imbalance between lipid anabolism and catabolism can result in abnormal lipid deposition and an increase in lipid metabolism syndromes. Such syndromes include metabolic dysfunction-associated steatotic liver disease (MASLD) [20,21], lipid metabolism disorders, and atherosclerotic cardiovascular disease (ASCVD) [22,23]. Consequently, elucidating the regulatory mechanisms of lipid homeostasis may create a unique therapeutic window for treating lipid metabolism disorder-related diseases by correcting lipid metabolism.

Oxidative stress (OS) is defined as a significant increase in pro-oxidant enzyme activity within the body, leading to excessive free radical production and ultimately causing an imbalance between the oxidative and antioxidant systems. It has been associated with the onset of diseases such as lipid metabolism disorders, diabetes, and hypertension [24,25]. In the context of physiological conditions, the body’s oxidative defence system and antioxidant mechanisms are known to maintain a dynamic equilibrium, a state which is widely acknowledged to play a crucial role in ensuring the stability of the physiological environment [26]. The core components of the oxidative reaction system include highly reactive molecules such as reactive oxygen species (ROS), with superoxide anion (·O_2_^−^), hydroxyl radicals (·OH), and hydrogen peroxide (H_2_O_2_) being the primary constituents [27]. It is acknowledged that two antioxidant systems exist within the body: the enzymatic antioxidant system and the non-enzymatic antioxidant system. Catalase (CAT) is a pivotal antioxidant enzyme within the enzymatic system, which is capable of decomposing hydrogen peroxide produced by oxidative processes. Accumulation of ROS (reactive oxygen species) within the body, or decline in antioxidant levels, can render them ineffective at neutralising oxidative components. This, in turn, disrupts the body’s redox homeostasis, inducing further ROS accumulation. Excessive ROS has been demonstrated to trigger lipid oxidation reactions, thereby affecting their structure, function, and physicochemical properties [28]. During this process, lipids undergo a chain reaction of peroxidation under the action of oxygen free radicals, ultimately producing end-products such as malondialdehyde (MDA) [29]. Excessive MDA levels have been demonstrated to further reduce the expression of antioxidant enzymes within the enzymatic antioxidant system, thereby exacerbating oxidative stress responses [30]. Previous studies have shown that the levels of MDA in hepatocytes can serve as a reliable indicator of the body’s overall oxidative stress status [31]. The present study indicates that systemic oxidative stress is closely associated with the severity of lipid metabolism disorders. Furthermore, previous studies have reported that elevated oxidative stress levels in adipose tissue represent an early pathological factor in metabolic syndromes such as MASLD [32]. It is noteworthy that the regulation of the redox balance within the adipose microenvironment has the potential to emerge as a viable intervention target for the amelioration of metabolic syndromes associated with lipid metabolism abnormalities. In this study, 1,8-cineole was found to reduce PA-induced lipid droplet accumulation in HepG2 and AML-12 cells, decrease TG and TC expression, enhance antioxidant enzyme activity, and reduce MDA and ROS levels.

In the present study, the IC50 values of 1,8-cineole in HepG2 and AML12 cells were approximately 3119 μg/mL (20,220 μM) and 2500 μg/mL (16,208 μM), respectively, whereas the concentrations used in subsequent experiments (250–1000 μg/mL; 1621–6486 μM) were well below cytotoxic levels. Previous studies have similarly demonstrated that 1,8-cineole exhibits relatively low cytotoxicity in different cell models. Rodenak-Kladniew reported that significant antiproliferative effects of 1,8-cineole in HepG2 and other tumor cell lines were mainly observed at relatively high concentrations, suggesting substantial cellular tolerance to this monoterpene [33,34]. In addition, Sampath demonstrated that plant-derived monoterpenes can exert important biological activities at concentrations lower than those required to induce overt cytotoxicity [35]. Similar observations were also reported by Lu, who showed that the biological responses to essential oil monomers vary considerably depending on the cell type and experimental conditions [36]. Therefore, the concentrations selected in the present study are more likely to reflect pharmacological and hepatoprotective effects rather than nonspecific cytotoxicity, and the differential IC50 values further suggest that tolerance to 1,8-cineole may differ among cell types.

Disruption of adipose homeostasis, caused by an imbalance in lipid metabolism, also triggers a series of inflammatory cascade reactions. Specifically, the abnormal secretion of pro-inflammatory adipokines by adipocytes, in conjunction with inflammatory cell infiltration, collectively drives the pathological progression of adipose inflammation. It is important to note that the hypoxic microenvironment induced by pathological adipose tissue proliferation drives phenotypic transformation in adipocytes. This transformation is characterised by altered cellular composition, abnormal angiogenesis, dynamic remodelling of the extracellular matrix, and upregulation of inflammatory mediators. This pathological process is defined as adipose tissue remodelling (ATR) [37]. Within this pathological process, dysfunctional hypertrophic adipocytes not only excessively release adipokines but also recruit immune cells such as macrophages to the adipose tissue via chemokines, thereby transforming adipose tissue into a major production centre for systemic inflammatory mediators. Research indicates that the TLR4/MyD88/NF-κB signaling pathway is a crucial intracellular pathway involved in inflammation, apoptosis, and immune responses [38,39]. TLR4, a pivotal inflammatory receptor within the Toll-like receptor family, plays a pivotal role in immune regulation. The ability to recognise the extracellular regions of its ligands and complexes appears to be a key factor in the stimulation of high expression of MyD88, which in turn activates the downstream NF-κB molecular pathway. Activation of the NF-κB signalling pathway leads to the production and release of inflammatory cytokines such as IL-6, IL-10, and TNF-α, which in turn re-activate the TLR4/MyD88/NF-κB pathway, triggering an inflammatory cascade and forming a vicious cycle [40]. The findings of this study demonstrated that 1,8-cineole led to a reduction in IL-1β expression (*p* < 0.01), exhibiting an effect that was comparable to that of NAC. Furthermore, the study demonstrated a reversal of PA-induced upregulation of IL-6 and TNF-α (*p* < 0.01), yet no significant improvement was observed for IL-18. This phenomenon may be attributed to the requirement for IL-18 release to occur, which in turn necessitates the assembly of the NLRP3 inflammasome and the subsequent cleavage of Caspase-1 [41]. Furthermore, the observation that higher levels of IL-18 precursor proteins demand greater Caspase-1 activity lends further credence to this hypothesis [42]. Palmitic acid has been demonstrated to only mildly activate NF-κB and is incapable of providing sufficient Caspase-1 activity, resulting in no significant change in IL-18. Furthermore, 1,8-cineole has been demonstrated to suppress PA-induced TLR4 and MyD88 expression. Following the inhibition of TLR4 with TAK-242, there was an alleviation of cellular lipid metabolism disorders, oxidative damage, and inflammatory responses [43,44].

NF-κB is a core transcription factor that regulates inflammation, immune responses, and cell survival. Abnormal activation of NF-κB has been closely associated with various diseases. The classical pathway is mediated by the IKK complex (IKKα/β/γ), which phosphorylates IκB to induce nuclear translocation of NF-κB (e.g., p65) and activate proinflammatory genes (TNF-α, IL-1β, etc.) [45]. NF-κB has also been demonstrated to increase hepatic lipid deposition by suppressing the lipid storage regulator PPARγ and promoting SREBP-1c. The activation of NF-κB has been demonstrated to induce the release of TNF-α and IL-1β from adipocytes and macrophages, thereby enhancing lipolysis and free fatty acid release, which in turn exacerbates insulin resistance [46,47]. Furthermore, NF-κB-driven inflammation has been demonstrated to increase ROS expression, thus forming a vicious cycle of “oxidative stress-inflammation” [48]. The present study found that 1,8-cineole significantly reduced PA-induced phosphorylation of IκBα and p65. Inhibition of the NF-κB signalling pathway using the IκB inhibitor BAY 11-7082 has been shown to significantly improve lipid metabolism disorders, inflammatory responses, and oxidative stress in AML-12 cells.

Despite these findings, several limitations of the present study should be acknowledged. First, this study was limited to in vitro experiments using AML-12 and HepG2 cell models, which cannot fully replicate the complexity of hepatic physiology in vivo, including hepatic blood flow, systemic metabolism, tissue pharmacokinetics, and the bioavailability of 1,8-cineole. Therefore, the effects of 1,8-cineole on lipid metabolism in living organisms may differ from those observed under cell culture conditions. Second, although the concentrations of 1,8-cineole used in this study were below the cytotoxic threshold, the highest concentration tested (1000 μg/mL, approximately 6 mM) remains relatively high from a pharmacological perspective, and its clinical relevance following oral or inhalational exposure in humans remains uncertain [49]. Third, the PA-induced lipotoxicity model represents a simplified in vitro model of metabolic dysfunction-associated steatotic liver disease and cannot fully reproduce the complex pathological features of metabolic liver disease, including fibrosis, systemic insulin resistance, and gut–liver axis involvement. Finally, although inhibitor experiments supported the involvement of the TLR4/MyD88/NF-κB signaling pathway, it remains unclear whether this pathway represents the primary mechanism underlying the protective effects of 1,8-cineole or one of several parallel pathways potentially involved, such as AMPK, PPAR-α, and SIRT1 signaling. Future in vivo studies and multi-pathway mechanistic investigations are therefore warranted.

## 4. Materials and Methods

### 4.1. Materials

#### 4.1.1. Chemicals and Reagents

**Figure 10:**
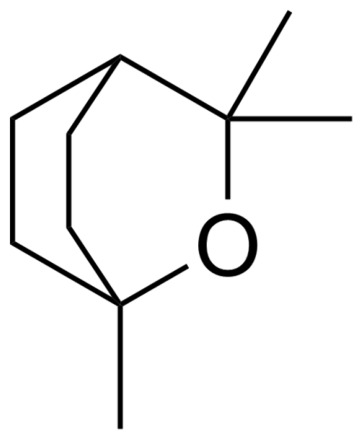
Chemical structure of 1,8-cineole.

1,8-Cineole (purity 99%) was obtained from Shanghai Macklin Biochemical Technology Co., Ltd. (Shanghai, China). The chemical structure of 1,8-cineole is presented in Figure 10. The human hepatocellular carcinoma cell line HepG2 and the mouse normal hepatocyte cell line AML-12 were procured from the Cell Bank of the Chinese Academy of Sciences (Shanghai, China). N-Acetyl-L-cysteine (NAC), BAY 11-7082 inhibitor, and TAK-242 inhibitor were procured from Shanghai Aladdin Biochemical Technology Co., Ltd. (Shanghai, China). The Oil Red O staining kit was procured from Beyotime Biotechnology (Shanghai, China). Assay kits for MDA, TC, CAT, and TG were procured from Nanjing Jiancheng Bioengineering Institute (Nanjing, China). Reactive oxygen species detection kits and Orlistat were procured from Beijing Solarbio Science & Technology Co., Ltd. (Beijing, China). Mouse TNF-α, IL-1β, and IL-6 enzyme-linked immunosorbent assay (ELISA) kits were procured from Shanghai Yanhui Biotechnology Co., Ltd. (Shanghai, China). The mouse IL-18 ELISA kit was procured from Wuhan Ruidi Biotechnology Co., Ltd. (Wuhan, China). The following antibodies were sourced from ABclonal Technology Co., Ltd. (Wuhan, China): NF-κB p65/RelA Rabbit mAb, Phospho-NF-κB p65/RelA-S536 Rabbit pAb, IκBα Rabbit pAb, Phospho-IκBα-S32 Rabbit mAb, TLR4 Rabbit pAb, MyD88 Rabbit pAb, and HRP-conjugated Goat anti-Rabbit IgG.

#### 4.1.2. Cell Lines and Culture Conditions

The human hepatocellular carcinoma cell line HepG2 and the mouse normal hepatocyte cell line AML-12 were obtained from the Shanghai Cell Bank of the Chinese Academy of Sciences. HepG2 cells were cultured in DMEM supplemented with 10% fetal bovine serum (FBS) and 1% penicillin–streptomycin. AML-12 cells were cultured in DMEM/F12 medium supplemented with 10% FBS and 1% penicillin–streptomycin. All cells were maintained at 37 °C in a humidified incubator containing 5% CO_2_.

#### 4.1.3. Main Instruments and Equipment

The main instruments used in this study included a Synergy H1 multifunctional microplate reader (BioTek Instruments Inc., Winooski, VT, USA); adjustable micropipettes (2, 10, 200, and 1000 μL; Eppendorf AG, Hamburg, Germany); a THZ-D thermostatic shaker (Shanghai Yiheng Scientific Instrument Co., Ltd., Shanghai, China); a Master Touch-R laboratory ultrapure water system (Shanghai Hitech Instruments Co., Ltd., Shanghai, China); a JEA2001 electronic balance (Shanghai Puchun Measuring Instrument Co., Ltd., Shanghai, China); a BSA223S analytical balance (Sartorius AG, Göttingen, Germany); a TD-600 low-temperature high-speed centrifuge (Sichuan Shuke Instrument Co., Ltd., Chengdu, China); an XB-70 ice maker (Ningbo Scientz Biotechnology Co., Ltd., Ningbo, China); a JY92-IIDN ultrasonic cell disruptor (Ningbo Scientz Biotechnology Co., Ltd., Ningbo, China); a SW-CJ-2FD clean bench (Yangzhou Chuangxin Medical Devices Factory, Yangzhou, China); an MVS-1 vortex mixer (Beijing Zijin Technology Development Co., Ltd., Beijing, China); a G154DWS autoclave (Zhicheng Instrument Co., Ltd., Shanghai, China); a Ts2FL research-grade inverted fluorescence microscope (Nikon Corporation, Tokyo, Japan); medical refrigerators (Qingdao Haier Co., Ltd., Qingdao, China); a Model 3308 CO_2_ incubator (Thermo Fisher Scientific, Waltham, MA, USA); an Amersham imaging system (GE Healthcare, Chicago, IL, USA); a Mini Trans-Blot electrophoretic transfer system (Bio-Rad Laboratories, Hercules, CA, USA); a Mini-PROTEAN Tetra electrophoresis system (Bio-Rad Laboratories, Hercules, CA, USA); and a PowerPac Basic power supply (Bio-Rad Laboratories, Hercules, CA, USA) and an upright fluorescence microscope (Motic China Group Co., Ltd., Xiamen, China).

### 4.2. Preparation of Experimental Reagents

1,8-Cineole was dissolved in DMSO to prepare a 300 mg/mL stock solution with a final DMSO concentration of 70% (*v*/*v*), followed by sterilization through a 0.22 μm filter. The stock solution was aliquoted and stored at −20 °C protected from light for no longer than 30 days. Before use, the stock solution was diluted with culture medium to the indicated concentrations.

10% BSA solution: Dissolve 0.9 g BSA powder in deionized water with heating at 60 °C until fully dissolved and clear. Adjust to the final volume to obtain a 10% (*w*/*v*) solution.

PA stock solution: Dissolve an appropriate amount of PA in 95 μL isopropanol using a water bath. Add 90 μL of this solution to 9 mL of 10% BSA solution and mix thoroughly to obtain a 2.5 mM PA stock solution.

Orlistat stock solution: Dissolve 0.5 mg Orlistat powder in 1 mL DMSO to prepare a 50 mg/mL stock solution.

NAC stock solution: Dissolve 81.6 mg NAC powder in 1 mL ultrapure water, followed by sonication to obtain a 0.5 M stock solution.

BAY 11-7082 stock solution: Dissolve 2 mg BAY 11-7082 powder in 965 μL DMSO to prepare a 10 mM stock solution.

TAK-242 stock solution: Dissolve 3.6 mg TAK-242 powder in 1 mL DMSO, followed by sonication to obtain a 10 mM stock solution.

### 4.3. Experimental Design and Experimental Procedures

#### 4.3.1. Cell Grouping

AML-12 cells were divided into the following groups: (1) Control group, cultured in complete medium for 48 h; (2) PA model group, treated with 400 μM PA for 48 h; (3) PA + 1,8-cineole groups, co-treated with 400 μM PA and different concentrations of 1,8-cineole for 48 h; (4) PA + Orlistat group, co-treated with 400 μM PA and 50 μg/mL Orlistat for 48 h; and (5) PA + NAC group, co-treated with 400 μM PA and 5 mM NAC for 48 h.

HepG2 cells were divided into the following groups: (1) Control group, cultured in complete medium for 48 h; (2) PA model group, treated with 400 μM PA for 48 h; (3) PA + 1,8-cineole groups, co-treated with 400 μM PA and different concentrations of 1,8-cineole for 48 h; and (4) PA + Orlistat group, co-treated with 400 μM PA and 50 μg/mL Orlistat for 48 h.

#### 4.3.2. MTT

Cells in the logarithmic growth phase were digested with trypsin and seeded into 96-well plates at a density of 1 × 10^4^ cells/mL (100 μL/well). The plates were incubated at 37 °C in a humidified atmosphere containing 5% CO_2_. After cell attachment, cells were treated for 48 h with complete medium containing either palmitic acid (PA, 100–500 μM, in 100 μM increments) or 1,8-cineole (250, 500, 1000, 2000, and 4000 μg/mL; corresponding to 1621, 3243, 6486, 12,972, and 25,944 μM).

1,8-Cineole stock solution (300 mg/mL) containing 70% DMSO was diluted directly into culture medium. The final concentrations of 1,8-cineole were calculated based on its molecular weight (154.25 g/mol) and are presented in both μg/mL and μM for clarity. The corresponding final DMSO concentrations (*v*/*v*) in each treatment group were 0.0583%, 0.1167%, 0.2333%, 0.4667%, and 0.9333% for 250, 500, 1000, 2000, and 4000 μg/mL (1621, 3243, 6486, 12,972, and 25,944 μM), respectively. The vehicle control group contained 0.9333% DMSO, matching the highest concentration group. A negative control group containing complete medium only was also included. Concentrations were unified to μM to ensure consistency in reporting between compounds.

Subsequently, 10 μL of MTT solution (5 mg/mL) was added to each well and incubated for 4 h. Then, 150 μL of DMSO was added to dissolve the formazan crystals, and the plates were shaken for 15 min. Absorbance was measured at 570 nm using a microplate reader, and relative cell viability was calculated.

#### 4.3.3. Oil Red O Staining

The detection of lipids was conducted in accordance with the established Oil Red O staining protocol. Samples in six-well plates were rinsed with ice-cold phosphate-buffered saline (PBS) and fixed with 4% paraformaldehyde for a period of 15 min. Following a brief rinse with 60% isopropanol for 20 s, the cells were stained with Oil Red O working solution in the dark for 30 min. Non-specific staining was eliminated through the process of differentiation with isopropanol for a duration of five seconds, subsequently followed by rinsing with ultrapure water. The distribution of red-stained lipid droplets was subsequently observed and analysed.

#### 4.3.4. Measurement of Oxidative Stress and Inflammation Markers

The measurement of TC, TG, MDA, and CAT levels was conducted using commercial biochemical assay kits. The intracellular levels of reactive oxygen species (ROS) were detected using the 2′,7′-dichlorodihydrofluorescein diacetate (DCFH-DA) fluorescent probe method. Levels of IL-6, TNF-α, and IL-1β were then quantitatively analysed using respective ELISA kits.

#### 4.3.5. Western Blot

The AML-12 and HepG2 cell suspensions were lysed on ice with 1 mL of strong lysis buffer, which contained protease and phosphatase inhibitors. Following sonication, the lysates were subjected to centrifugation at 12,000 rpm for a duration of 15 min at a temperature of 4 °C. Thereafter, the resultant supernantants were collected and stored for subsequent analysis. The protein concentration in the superior aspect of the mixture was determined by means of a BCA protein assay kit. Proteins were denatured by subjecting them to boiling for a period of 20 min, after which they were stored at a temperature of −20 °C for subsequent use. The protein samples were loaded onto 10% SDS-PAGE gels, with the quantity of protein applied to each gel being equivalent. Electrophoresis was performed initially at 80 volts for 30 min, followed by 120 volts until completion. Subsequently, proteins were transferred to PVDF membranes at 200 mA for a period of 2 h. The membranes were blocked at room temperature with fast protein-free blocking solution for a period of 90 min, with gentle shaking, followed by three 5-min washes with PBST. The membranes were then subjected to an overnight incubation at 4 °C, with gentle shaking, in primary antibodies at the following dilutions: P65 (1:5000), IkBα (1:5000), Phospho-IkBα (1:1000), Phospho-P65 (1:5000), TLR4 (1:1000), and MyD88 (1:1000). Following three 5-min washes with PBST, the membranes were then subjected to an incubation with HRP-conjugated secondary antibody (1:8000) at room temperature for a period of two hours, with gentle shaking. Following a series of three final washes with PBST, the protein bands were visualised using a multi-functional imaging system.

#### 4.3.6. Statistical Analysis

All experiments were repeated independently at least three times. The data are presented as the mean ± standard deviation (Mean ± SD). The quantification of band intensities from Western blot analyses was conducted utilising Image J software (Version 1.54p, National Institutes of Health, Bethesda, MD, USA). The raw data were processed and visualised using GraphPad Prism 9.0 software, and the scientific figures were finalised and optimised using Adobe Illustrator (Version 28.0, Adobe Inc., San Jose, CA, USA) for vector graphics. The statistical analysis was conducted utilising SPSS 21.0 software. One-way analysis of variance (ANOVA) was employed for the purpose of conducting comparisons among multiple groups. A significance level of α = 0.05 (95% confidence interval) was established. Statistical significance was attributed to differences when *p* < 0.05.

## 5. Conclusions

**Figure 11:**
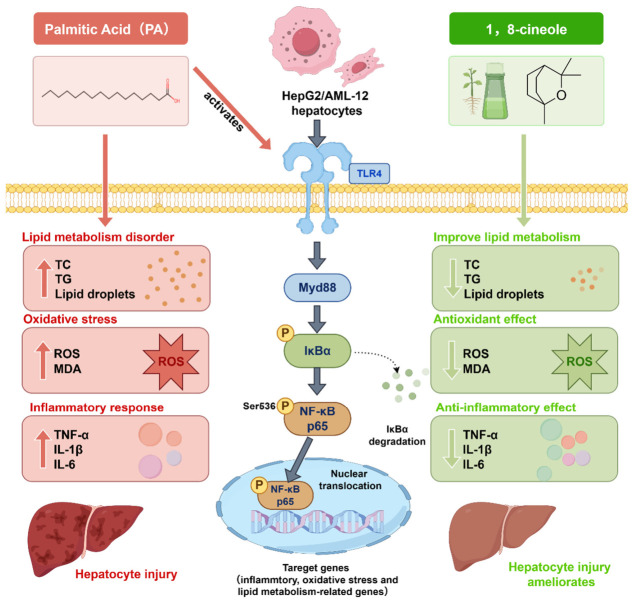
Proposed mechanism underlying the protective effects of 1,8-cineole against PA-induced hepatocyte injury. Red lines indicate inhibitory effects, whereas green lines indicate alleviating/protective effects. TC, total cholesterol; TG, triglycerides; ROS, reactive oxygen species; and MDA, malondialdehyde.

The present study demonstrates that 1,8-cineole significantly reduces total cholesterol (TC) and triglyceride (TG) levels in palmitic acid (PA)-induced high-fat models of HepG2 and AML-12 cells, effectively improving lipid metabolism disorders. Concurrently, 1,8-cineole treatment led to a significant decrease in reactive oxygen species (ROS) levels and malondialdehyde (MDA) content in AML-12 cells, thereby mitigating oxidative stress-induced damage. Furthermore, the observed reduction in pro-inflammatory mediators, including tumor necrosis factor-α (TNF-α), interleukin-1β (IL-1β), and interleukin-6 (IL-6), resulted in a corresponding alleviation of cellular inflammatory responses. The molecular mechanism studies revealed that 1,8-cineole has the capacity to downregulate the expression levels of key proteins, including Toll-like receptor 4 (TLR4), myeloid differentiation factor 88 (MyD88), and nuclear factor κB (NF-κB). The efficacy of TLR4 inhibitors and NF-κB pathway inhibitors serves to confirm the pivotal role of the TLR4/MyD88/NF-κB signaling pathway in inflammation, oxidative stress, and lipid metabolism disorders. In summary, 1,8-cineole has been demonstrated to effectively ameliorate oxidative stress, inflammatory responses, and lipid metabolism disorders in hepatocytes by inhibiting the TLR4/MyD88/NF-κB signaling pathway. The proposed mechanism underlying the protective effects of 1,8-cineole is illustrated in Figure 11.

PA activates the TLR4/MyD88/NF-κB signaling pathway, leading to NF-κB p65 phosphorylation and nuclear translocation, which subsequently promotes the expression of genes associated with inflammation, oxidative stress, and lipid metabolism disorders. As a result, intracellular lipid accumulation, reactive oxygen species (ROS) generation, malondialdehyde (MDA) production, and pro-inflammatory cytokine levels (TNF-α, IL-1β, and IL-6) are increased, ultimately contributing to hepatocyte injury. Treatment with 1,8-cineole suppresses activation of the TLR4/MyD88/NF-κB signaling pathway, thereby alleviating oxidative stress, inflammatory responses, and lipid metabolism disorders in hepatocytes.

## Data Availability

The original contributions presented in this study are included in the article. Further inquiries can be directed to the corresponding authors.
